# A Home Hazard Removal Program to Reduce Falls in Community-Dwelling Older Adults

**DOI:** 10.1093/geroni/igab046.1486

**Published:** 2021-12-17

**Authors:** Susy Stark

**Affiliations:** Washington Unversity, St Louis, Missouri, United States

## Abstract

The majority of falls experienced by older adults occur in the home with home hazards associated with an increased risk of falling. Low-income older adults, who have more disability and live in substandard housing, need feasible interventions to help them safely age in place. The Home Hazard Removal Program (HARP) is a new home hazard removal and fall risk self-management program delivered in the home by occupational therapists to prevent falls. To evaluate the program, a randomized control trial was conducted with 310 community-dwelling older adults receiving aging services in the community. HARP had high acceptability with older adults and was feasible to deliver in the community. Adjusted for fall risk, individuals in the HARP group fell 1.4 times versus 2.2 times in the control group over 12 months. This low-cost home hazard removal program demonstrated acceptability, feasibility, and a significant reduction in falls for at-risk community-dwelling older adults.

